# Structural and functional radiomics for lung cancer

**DOI:** 10.1007/s00259-021-05242-1

**Published:** 2021-03-11

**Authors:** Guangyao Wu, Arthur Jochems, Turkey Refaee, Abdalla Ibrahim, Chenggong Yan, Sebastian Sanduleanu, Henry C. Woodruff, Philippe Lambin

**Affiliations:** 1 The D-Lab, Department of Precision Medicine, GROW–School for Oncology, Maastricht University Medical Centre+, 6229 Maastricht, The Netherlands; 2 Department of Radiology, Union Hospital, Tongji Medical College, Huazhong University of Science and Technology, Wuhan, China; 3 Hubei Province Key Laboratory of Molecular Imaging, Wuhan, China; 4 Department of Diagnostic Radiology, Faculty of Applied Medical Sciences, Jazan University, Jazan, Saudi Arabia; 5 Department of Radiology and Nuclear Medicine, GROW – School for Oncology, Maastricht University Medical Centre+, Maastricht, The Netherlands; 6 Division of Nuclear Medicine and Oncological Imaging, Department of Medical Physics, Hospital Center Universitaire De Liege, Liege, Belgium; 7 Department of Nuclear Medicine and Comprehensive Diagnostic Center Aachen (CDCA), University Hospital RWTH Aachen University, Aachen, Germany; 8 Department of Medical Imaging Center, Nanfang Hospital, Southern Medical University, Guangzhou, China

**Keywords:** Lung cancer, Radiomics, Artificial intelligence, Medical imaging

## Abstract

**Introduction:**

Lung cancer ranks second in new cancer cases and first in cancer-related deaths worldwide. Precision medicine is working on altering treatment approaches and improving outcomes in this patient population. Radiological images are a powerful non-invasive tool in the screening and diagnosis of early-stage lung cancer, treatment strategy support, prognosis assessment, and follow-up for advanced-stage lung cancer. Recently, radiological features have evolved from solely semantic to include (handcrafted and deep) radiomic features. Radiomics entails the extraction and analysis of quantitative features from medical images using mathematical and machine learning methods to explore possible ties with biology and clinical outcomes.

**Methods:**

Here, we outline the latest applications of both structural and functional radiomics in detection, diagnosis, and prediction of pathology, gene mutation, treatment strategy, follow-up, treatment response evaluation, and prognosis in the field of lung cancer.

**Conclusion:**

The major drawbacks of radiomics are the lack of large datasets with high-quality data, standardization of methodology, the black-box nature of deep learning, and reproducibility. The prerequisite for the clinical implementation of radiomics is that these limitations are addressed. Future directions include a safer and more efficient model-training mode, merge multi-modality images, and combined multi-discipline or multi-omics to form “Medomics.”

## Introduction

Lung cancer ranks first in cancer-related deaths and second in the new cancer cases in both males and females as reported by the American Cancer Society in 2020 [1]. The 5-year survival rate ranges from 5% for patients with metastatic disease to 57% when lung cancer is diagnosed and treated at an early stage [2]. Unfortunately, most patients with lung cancer only have mild clinical symptoms at an early stage, but the symptoms appear when the cancer is at an advanced stage [3].

Both the US-based national lung screening trial (NLST) and the Dutch-Belgian lung cancer screening trial (NELSON) concluded that the use of low-dose computed tomography (LDCT) for high-risk lung cancer populations decreases lung cancer mortality up to 60% in certain subpopulations, due to early detection and management [4, 5]. A substantial number of early-stage lung cancer patients have been identified using LDCT lung cancer screening [6]. The main goals of precision medicine research in lung cancer could be generally categorized into early-stage detection and/or diagnosis and highly tailored treatment and care in the advanced stage.

The use of radiologic images in medicine has become crucial in clinical practice, for both oncologic and non-oncologic cases. In oncology, medical imaging is used for every aspect of patients’ management, including screening, diagnosis, treatment, and prognosis assessment of the disease. Over the past few decades, modern medical imaging has progressed from single structural imaging to combination of functional imaging [7]. Structural imaging refers to methods used to both visualize and evaluate anatomical details, and functional imaging is used to assess the physiology and molecular processes of tissues and organs [8]. Evidence has shown that the relationship between the structure and function of the lung is imperfect, which means that structural and functional images could have some common biomarkers, but certainly have independent biomarkers [9]. However, the correspondence between structural and functional images in the field of lung cancer still remains to be elucidated.

**Fig. 1:**
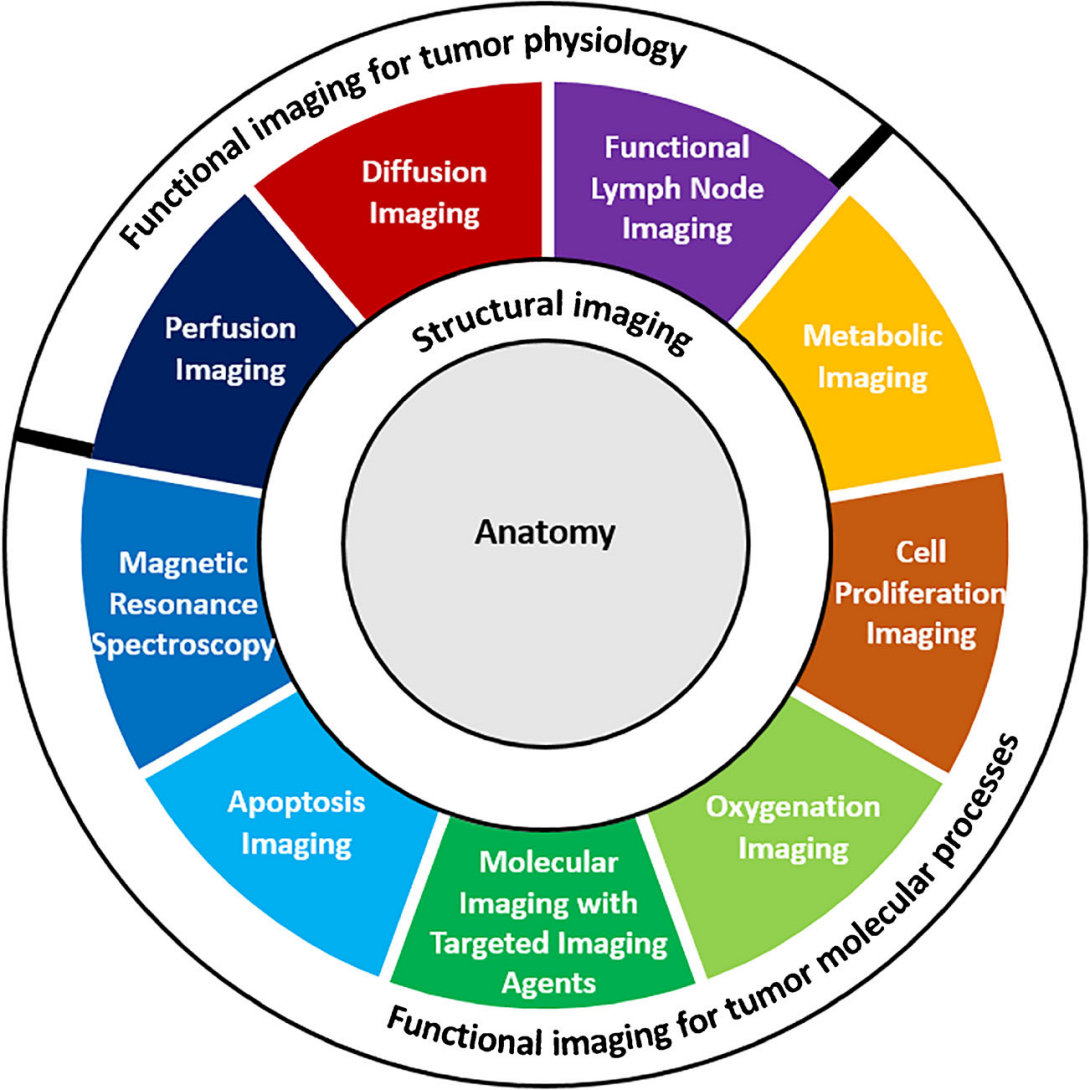
Composition of functional and structural imaging for tumors. Structural imaging refers to techniques, which are used to visualize and analyze the anatomical information of the human body. Functional imaging refers to approaches that are the study of tumor physiology and molecular process

Radiomics refers to the extraction and analysis of quantitative image features from medical images using mathematical and machine learning methods to explore possible ties with biology and clinical outcomes [10]. Radiomic features extracted from structural and functional images (as summarized by Torigian et al. with detailed imaging modalities) can separately reflect the anatomical and functional information of the lesions [11] (Fig. 1). These features, mostly invisible to the unaided eye, have the potential to reduce the workload of clinicians and to increase the quality of diagnosis, prognosis, and treatment. The ultimate goal of radiomics is to build quicker and more reliable clinical decision support systems to assist clinicians rather than replacing them [12].

In this review, we present the development of radiologic features, from semantic and handcrafted radiomic to deep radiomic features, from the perspective of clinicians. In addition, we summarized the latest applications of structural and functional radiomics on early and advanced-stage lung cancer. Furthermore, we address the possible limitations of radiomics and set out future directions with respect to lung cancer.

## Development from semantic to radiomic features

### Semantic and clinical features

**Fig. 2:**
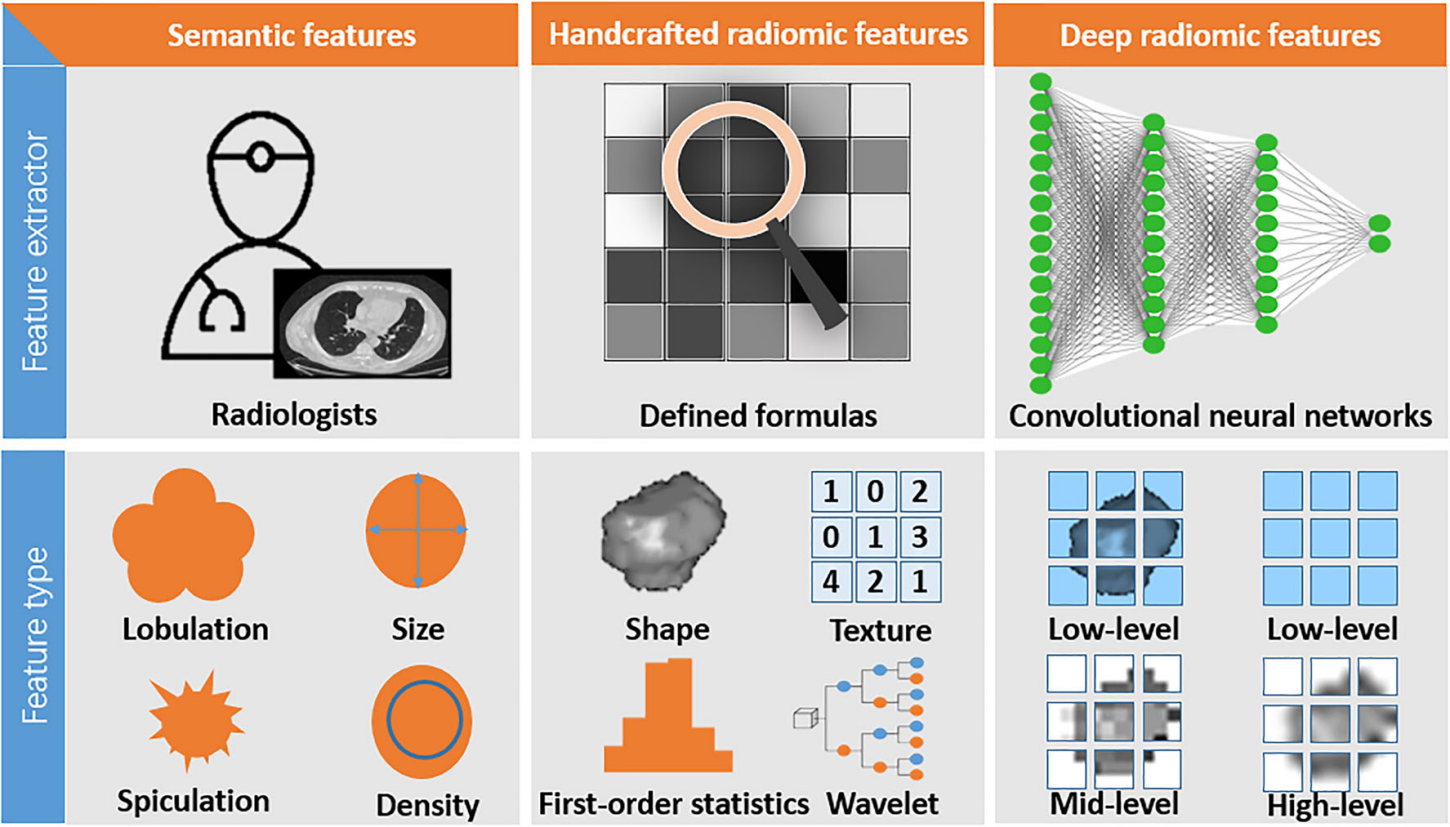
A comparison of semantic, handcrafted radiomic, and deep radiomic features

Imaging techniques have been widely used in clinical practice for different purposes in lung cancer. These techniques include radiography, CT, MRI, and PET/CT. Radiologists analyze the images to detect lesions, and then use a set of qualitative (e.g., shape, location, speculation, and lobulation) and quantitative (e.g., size, volume, density, signal, and standardized uptake values (SUVs)) features to describe and analyze lesions (Fig. 2). Radiologists have been seeking to identify specific signs from images that can be used to determine the pathological type, degree of malignancy, and prognosis of cancer.

However, this approach involves a wealth of experience leading to subjective discrepancies. For this reason, the Fleischner Society has provided a series of glossaries and recommendations for describing and measuring thoracic images [13, 14]. Based on the evidence that semantic CT features have prognostic value, the Lung Imaging Reporting and Data System (Lung-RADS) has been developed to improve the interpretations and understanding of lung cancer screening CT and promote management [15]. Furthermore, the Response Evaluation Criteria in Solid Tumors (RECIST 1.1), a special version (iRECIST) for assessing response to immunotherapy, and a version for PET (PERCIST) have been successfully validated in clinical trials [16–18].

Clinical features have showed additional prognostic utility when combined with semantic CT characteristics, which could be used to construct a clinical model for predicting the risk of malignancy, invasiveness, and poor prognosis of lung cancer [19–21]. A generally recognized and validated clinical model for the malignancy risk of solitary pulmonary nodule is the Brock/PanCan model, which includes both clinical (age, sex, and family history of lung cancer) and semantic CT (emphysema, spiculation, size, type, location, and nodule count) features [22]. However, the subjective nature of these metrics can theoretically restrict the consistency of models based on semantic features [23].

### Handcrafted radiomic features

Handcrafted radiomic features are the result of mathematical formulas that take image pixel values from within a region of interest (ROI) as an input and output a number, the so-called feature value, a process that can be automated. In order to quantitatively and automatically identify and interpret imaging findings, these handcrafted radiomic features can be used as a machine learning framework to correlate them with the underlying biology and clinical outcomes (Fig. 2) [24, 25]. The classical radiomic workflow involves image acquisition, lesion segmentation, pre-processing, feature extraction, feature selection, modeling, model validation/evaluation, and if the last step proves successful, clinical implementation.

Strict quality management at each step is necessary to ensure applicability and generalization of the developed model, and the first step is no exception as high-quality images are of supreme importance in a radiomics study. In the following task, experienced radiologists often manually delineate the images slice by slice to define the ROI. Manual delineation, however, is time consuming and vulnerable to inter-observer variability [26]. Advanced semi-automatic or fully automatic segmentation can enhance the repeatability of radiomic features [27].

Pre-processing techniques (e.g., resampling, denoising, histogram equalization and image normalization) can be used to minimize the variance among images when various scanners or scanning and reconstruction parameters are used [28, 29]. For feature extraction, McNitt-Gray et al. summarized several software or packages that can automatically perform this task, producing first-order statistics, shape, texture, and filter based features [30]. The input format (file type and modality) and the type of features should also be taken into consideration during this processing. Of note, radiomic features are sensitive to differing acquisition and reconstruction parameters employed by the multiple centers, and harmonization in the image domains or feature domains are being explored [31].

The selection of stable, important, and predictive features is another significant step in the radiomics pipeline. There are three methods to perform feature selection: (i) filter methods select features independently from the model, often using statistical tests and correlations of features with the outcome, such as Chi-square, Mann-Whitney, and Pearson’s correlation. (ii) wrapper methods generate different subsets of features that are used to train multiple models, which are ranked based on model performance. (iii) embedded methods perform feature selection and model training simultaneously, with the least absolute shrinkage and selection operator (LASSO) method being the most widely published [32]. The choice of feature selection method also depends on which machine learning algorithm is chosen for the next step.

Once the features that will serve as input to the model have been selected, many machine learning algorithms are available to train classification or regression models. These algorithms can be broadly classified into supervised (e.g., logistic regression, support vector machine, random forest, and artificial neural network) and unsupervised (e.g., clustering and auto-encoder) solutions. In short, supervised methods optimize performance by updating the inner model configuration based on the desired outcome, while unsupervised methods rely on patterns in the predictors, without having access to the actual outcome. The composition, consistency, and interpretability of data are considerations that need to be weighed when choosing models. External validation is required to properly assess efficiency and generalization. Many tools from data science are used to evaluate the performance of a trained model, such as the receiver operating characteristic (ROC) or precision recall curve, area under the ROC curve (AUC), concordance index (C-index), confusion matrix, calibration, and decision curve analysis [33]. Multivariate Cox regression and Kaplan-Meier curve are popular tools for survival analysis [34]. When using these metrics, the balance of data, i.e., the number of cases in each class, needs to be carefully considered [35]. There are a number of methods to artificially balance datasets as Fotouhi et al. summarized, especially for classification problems, such as oversampling, undersampling, synthetic minority over-sampling technique (SMOTE), and adaptive synthetic (ADASYN) techniques [36].

### Deep radiomic features

Deep learning is a broader family of machine learning methods inspired by our brain’s own network of neurons [37]. The convolutional neural network (CNN) is commonly used in the analysis of images and has found its way to the field of medical imaging. The term deep comes from the large number of various types of layers (interconnected “slices” of the network): convolution, pooling, activation, and full connection (Fig. 2). In convolution layers, convolutional kernels slide over the image to automatically extract and select features that can be named deep radiomic features from a tailored and well-trained CNN structure (e.g., AlexNet, VGG, ResNet, Inception, and DenseNet) [38].

Handcrafted and deep radiomic features have many similarities but are also distinct. Deep radiomic features in shallow layers define intensity, shape, and texture details that are similar to handcrafted radiomic features; as the layers get deeper, more and more abstract features are extracted that are learnt from exposure to data, making these features difficult to interpret [39]. This makes a model based on deep radiomic features look like a black box, where the connection between the input and output is not understood. However, if the interpretability of the model is not considered to be of critical relevance, deep radiomic features are an effective supplement for handcrafted radiomic features, since deep learning networks can take whole images as the input, making them independent of ROI segmentation and allowing for added features such as anatomical location. In addition, trained deep learning models and learned features can be adapted to other imaging tasks using transfer learning [40].

Of note, the medical machine-learning field, unlike the conventional clinic or pharmacy, does not have a standardized research methodology. As the field grows the need for standardized methodology increases for study comparability. Methodological recommendations for the prediction or radiomic analysis are helpful in the creation and evaluation of clinical performance [10, 41, 42]. However, given that the field is new and rapidly changing, constant updates and additions to guides are required.

## Applications of structural radiomic features in lung cancer

Chest radiography is an initial screening technique for lung cancer due to its low cost and convenience. A deep learning detection algorithm showed a high sensitivity for pulmonary nodules and lung cancer in the NLST and private datasets, and these studies suggested that this technique, as a second reader, could enable radiologists to better detect pulmonary nodules and lung cancer. [43–46]. In addition, Lu et al., developed a model focused on deep learning to classify a population of smokers with high-risk of lung cancer and the AUC was slightly higher than the eligibility requirements for clinical assessment (0.76 vs. 0.63) [47]. LDCT can detect a small lesion to provide more information than a radiograph. Using deep learning with LDCT, a 90.0% sensitivity for pulmonary nodule detection has been achieved [48].

With the large numbers of small nodules found on LDCT lung cancer screening, an instrument for the classification of malignant nodules would boost clinical management. The performance of a CNN (AUC = 0.90) outperformed the Brock (AUC = 0.87) model for estimating the likelihood of malignant nodules [49]. Garau et al. found that the handcrafted radiomics features model had a higher AUC than the Lung-RADS clinical model (0.86 vs. 0.76) in the external validation [50]. In the case of solitary noncalcified nodules, the radiomic model incorporating perinodular and intranodular features demonstrated improved performance (intranodular radiomic features: AUC of 0.75; combination: AUC of 0.80) in distinguishing adenocarcinomas from granulomas in the non-contrast chest CT [51]. Some investigators, on the other hand, observed that the accuracy of the radiomics model was close to radiologists when contrast CT was used [52]. In addition, MRI radiomics also demonstrated strong success (AUC = 0.88) in the differentiation of lung malignancies and benign lesions [53]. A follow-up scan is a recommended method for the management of accidental pulmonary nodules. The changes of features (delta-radiomic) on the baseline and follow-up scans were able to be used to predict malignancy of nodules, and the dynamic details (e.g., tumor doubling time and growth) can be predicted by baseline scan [54–56].

In regard to the classification of histological subtypes, the combination of handcrafted radiomic and clinical features of a logistic regression nomogram was used to categorize small cell lung cancer (SCLC) and non-small-cell lung cancer (NSCLC) with an AUC of 0.94 and an accuracy of 86.2% [57]. In addition, the study selected five handcrafted radiomic features as a signature for differentiating lung squamous cell carcinoma (SCC) from adenocarcinoma with an AUC of 0.89 [58]. Research involving 920 patients showed that both handcraft (AUC of 0.79) and deep (AUC of 0.84) radiomics could attain high performance in distinguishing adenocarcinoma, SCC, and SCLC [59]. Recent studies have documented that a radiomic signature can provide information on the level of Ki-67 expression [60].

The majority type of early lung cancer is adenocarcinoma, in which adenocarcinoma is in situ (AIS) or minimally invasive adenocarcinoma (MIA) has a near 100% 5-year survival probability after resection [61]. Recently, several studies have documented that CT-based handcrafted and deep radiomics have been able to determine the invasiveness of lung adenocarcinoma manifesting as sub-solid and solid nodules with AUC of 0.77 to 0.90 [62–64]. The combination of other variables such as clinical-, semantic-, and intraoperatively pathological features can boost the accuracy of final pathology [65, 66]. In addition, a fusion of intranodular (solid and ground-glass) and perinodular radiomic features can be more predictive than the full gross tumor alone [67, 68]. In response to the specific types of invasive adenocarcinoma, both handcrafted and deep radiomics have shown efficacy in predicting higher invasive levels of solid/micropapillary adenocarcinoma [69–71].

Early-stage lung cancer, adenocarcinoma in particular, spreads through intrusive neighboring lymphovassel, pleura, and air space, which impairs the surgical result and affects the surgical approach. Two-dimensional texture features can individually predict lymphovascular invasion with an AUC of 0.86 [72]. Some researchers proposed that radiomic signature could potentially help to identify the pleural invasion of stage I pulmonary adenocarcinoma [73]. In addition, Zhuo et al. combined radiomic and semantic features (the size of the solid component and mediastinal lymphadenectasis) with an AUC of 0.99 for the prediction of the existence of spread through air space [74]. Intraoperative lymph node status is critical to choose a systematic or selective lymph node dissection. Several studies have shown that handcrafted and deep radiomic features of the intra/peri-tumor can be used as biomarkers to predict lymph node metastases [75–77]. Furthermore, in the case of pleural metastases, radiomic features may have a diagnostic power with AUC of 0.93 [78].

With the development of personalized treatment for lung cancer, the identification of therapeutically actionable mutations ((e.g., Epidermal growth factor receptor (EGFR), anaplastic lymphoma kinase (ALK), programmed cell death 1 ligand, (PD-L1), and v-Raf murine sarcoma viral oncogene homolog B1 (BRAF)) has been a significant premises for an optimal treatment strategy. Thanks to the existence of non-invasive, simple, and low cost of radiomics compared to gene detection, which has demonstrated strong predictive efficacy for the mutation type and can used as an alternative method [79–82]. In addition to predicting the status of gene mutations, some studies aimed to directly predict the treatment response, such as Immunotherapy, chemotherapy, and radiotherapy [83–86]. Another study has been able to predict biological substrates, such as tumor hypoxia with radiomics [87]. Dercle et al. conducted prospective and retrospective experiments in multicenter clinical trials specifically to predict the susceptibility of lung cancer to nivolumab, docetaxel, and gefitinib with an AUC of 0.77, 0.67, and 0.82, respectively [83]. Radiomics has been proven to be a valuable method in radiotherapy preparation, radiotherapy response, pathologic response to neoadjuvant chemoradiotherapy, and side effects of radiation pneumonitis [84–86].

Long-term prognostic outcomes (e.g., overall survival, disease-free survival, distant metastasis, and local recurrence) after therapy are chronically tracked and are expected to be primarily dependent on therapeutic, pathological, and histological details. Radiomics can extract useful and unknown image features for predicting prognosis prior to treatment. Choe et al. observed that handcrafted radiomic features provided additional prognostic benefit outside the clinical-pathologic model alone (AUC: 0.78 vs. 0.73) [88]. In addition, radiomics has demonstrated a certain capacity to predict recurrence (AUC of 0.74–0.76) and distant metastasis (AUC of 0.89) [89, 90]. However, most studies have reported mild or even negative prognostic [91–93].

## Applications of functional radiomic features in lung cancer

The most common functional imaging for lung cancer is 18F-FDG PET, which can reflect tumor glucose metabolism and capture both metabolic and structural information when paired with CT (PET/CT). Functional imaging has been widely used in clinical practice for diagnosis, staging, genetic mutation estimation, treatment response evaluation, and prognostic assessment.

Some studies have used PET-based radiomics alone to forecast clinical outcomes [94–97]. A research with 210 adenocarcinoma and 186 SCC patients showed that a PET-based radiomics signature could distinguish lung adenocarcinoma from SCC, albeit without external validation [94]. In 264 NSCLC patients, Tau et al. used PET images to train a CNN to predict lymph node metastases (accuracy of 80%) or distant metastases (accuracy of 63%) with moderate performance [95]. Some studies indicated that both PET-based radiomic features provided the prediction of prognostic outcome for lung cancer patients with radiotherapy or chemo-radiotherapy, whereas semiquantitative PET factors were not available [96]. Both PET-based handcrafted and deep features can predict the response of immunotherapy in lung adenocarcinoma [97, 98].

PET/CT provides more precise location information and more detailed surrounding structures than PET alone, meaning that radiomics based on PET/CT is able to extract both functional and structural feature and has a wider application prospect than PET or CT alone. One of the applications of diagnosis is using radiomics to distinguish pulmonary tuberculosis, lymphoma, and other benign lesions from lung cancer [99–101]. Another application of diagnosis based on PET/CT radiomics is to distinguish lung adenocarcinoma from SCC as well as primary from metastatic lung cancer [102, 103]. A recent study with a small sample size (with 91 patients) attempted to use 2 PET and 2 CT features for the identification of growth patterns in early lung invasive adenocarcinoma [104].

Furthermore, the prediction of EGFR mutations is a representative example of application of radiomics based on PET/CT. In a study with 248 lung cancer patients without treatment, researchers found that their model for prediction of EGFR mutations could reach an AUC of 0.87 when combined clinical and radiomic signature [105]. Similar performance has also been reported in another retrospective study [106]. In addition, for patients with EGFR mutation, a deep radiomic score was a non-invasive tool to identify NSCLC patients susceptible to tyrosine kinase or immune checkpoint inhibitors [107].

Yang et al. concluded that a radiomic nomogram based on PET/CT rad-core and clinicopathological features was able to predict the overall survival of NSCLC patients [108]. A study focused on prediction of prognosis after immunotherapy suggested that PET/CT-based radiomics signature could be used before the start of treatment to identify those most likely to benefit from immunotherapy for advanced NSCLC patients [109]. In addition, a multicenter study with 87 early stage NSCLC patients underwent radiotherapy selected one PET and one CT feature to predict local recurrence and reached good performance (100% sensitivity and 96% specificity) [110]. Compared to handcrafted radiomic features, deep radiomic features had a significantly better prognostic value [111].

Dual-energy CT (DECT) provides additional perfusion information of tumor using quantification of iodine enhancement at different phases. In 93 lung cancer patients with examination of DECT, entropy from iodine overlay maps enhanced prediction of overall survival to pathological stage alone (C-index, 0.72 vs. 0.67) [112]. Table 1 summarized the radiomic studies using both structural and functional images.

**Table 1:** Summary of some radiomic studies using both structural and functional images

Study	Study design	Modality	Population	Features type	Features selection	Model algorithm	Type of validation	Outcome
Du et al. (2020)	Retrospective Single-center	PET/CT	77 Tuberculosis 79 Lung cancers	Clinical Semantic Handcrafted radiomic	LASSO	LR	Leave one out Without external validation	Diagnosis
Sibille et al. (2020)	Retrospective Single-center	PET/CT	302 lung cancer 327 lymphoma	Deep radiomic	CNN	CNN	Leave one out Without external validation	Diagnosis
Kang et al. (2019)	Retrospective Single-center	PET/CT	157 malignant 111 benign patients	Clinical Handcrafted radiomic	LASSO	LR	Bootstrapping validation Without external validation	Diagnosis
Han et al. (2020)	Retrospective Single-center	PET/CT	867 adenocarcinomas 552 SCCs	Handcrafted radiomic Deep radiomic	Ten feature selection methods	10 ML models and the VGG16	Leave one out Without external validation	Diagnosis
Kirienko et al. (2018)	Retrospective Single-center	PET/CT	534 Lung lesions	Handcrafted radiomic	–	LDA	Leave one out Without external validation	Primary or metastatic lung lesions
Shao et al. (2020)	Retrospective Single-center	PET/CT	91 GGNs	Semantic Handcrafted radiomic	LASSO	LR	Bootstrapping validation Without external validation	Lepidic or acinar-papillary growth
Zhang et al. (2020)	Retrospective Single-center	PET/CT	248 NSCLCs	Clinical Handcrafted radiomic	LASSO	LR	Leave one out Without external validation	EGFR mutation
Liu et al. (2020)	Retrospective Single-center	PET/CT	148 Adenocarcinomas	Handcrafted radiomic	RF/LR	Xgboost	Leave one out Without external validation	EGFR mutation
Mu et al. (2020)	Retrospective Multi-center	PET/CT	681 NSCLCs	Deep radiomic	CNN	CNN	Leave one out With external validation	EGFR mutation Treatment response
Yang et al. (2020)	Retrospective Single-center	PET/CT	315 NSCLCs	Clinical Handcrafted radiomic	LASSO	LR	Leave one out Without external validation	Survival
Mu et al. (2019)	Retrospective/ prospective Single-center	PET/CT	194 Stage IIIB-IV NSCLCs	Clinical Handcrafted radiomic	LASSO	LR	Leave one out With external validation	Survival after immunotherapy
Dissaux et al. (2020)	Retrospective Multi-center	PET/CT	87 Early-stage NSCLCs	Clinical Semantic Handcrafted radiomic	Univariate/ Multivariate analysis	Cox	Leave one out With external validation	Local Recurrence after radiotherapy

*SCC*, squamous cell carcinoma; *NSCLC*, non-small cell lung cancer; *GGN*, ground-glass nodule; *LASSO*, least absolute shrinkage and selection operator; *CNN*, convolutional neural network; *RF*, random forest; *LR*, logistic regression; *ML*, machine learning; *EGFR*, epidermal growth factor receptor

## Limitations and challenges

While radiomics has been successfully applied in the quantitative analysis of structural and functional images of lung cancer, certain limitations and obstacles must be faced and resolved before it is implemented in clinical practice. The first is that radiomics is a data-hungry approach. Large, diverse, multicenter, and high-quality data is needed to generalize the results and conclusions of radiomics studies. The creation of vast databases of medical images is currently problematic, mostly because sharing or exchange of data between hospitals and institutes is insufficient and the processing of data collection is time consuming. However, data sharing could pose possible ethical and legal dangers. Furthermore, the imaging data needs to be labeled with correct outcomes in order to be used for training, a process that adds to the cost burden. As the golden standard, the labels are also strongly contingent on the experience of physicians, subjective and complex [113]. Histopathological observations, for example, can be constrained by sampling errors and observer heterogeneity. Unsupervised and self-supervised methods are independent of particular labels, though accuracy and interpretability are sacrificed [114]. Precise segmentation is essential for handcrafted radiomics to select areas of interest for tumors. Manual segmentation is susceptible to inconsistency from multiple readers and time consuming. While automatic and semi-automatic segmentation has been used to increase objectivity and minimize time costs, there is no norm for guiding or assessing segmentation efficiency. Deep radiomics do not need explicit segmentation, although a rough ROI selection is required to conserve computing resources and reduce the effect of noise.

AI algorithms have made great strides in recent years, but the road is long and tortuous. There are plenty of methods to choose from during the process of preprocessing, feature extraction, feature selection, and modeling, though standard and appropriate workflow or guidelines for methodology and evaluation system are still uncommon. Currently, the most effective algorithm is deep learning based on artificial neural network that produce a huge number of computational parameters and requires high-performance computers to provide processing power and hardware support. More powerful and intelligent algorithms will certainly appear in the future, but the timing of the emergence may depend greatly on development of neuroscience and computer science. Data-driven radiomics especially deep radiomic features and models are basically a set of data that transforms into black boxes without intuitive interpretability. These models may have an even higher performance than humans, but they are unacceptable to clinicians who do not grasp how the machine makes decisions and works.

Furthermore, reproducibility is a basic requirement for clinical use. The variation in image acquisition and reconstruction as well as in the radiomics process can influence the reliability of the features, which is why many models do not perform well on independent external validation datasets. Although some harmonization approaches can reduce the batch effects in multicentric studies, the need for a “reference batch” to calibrate the harmonization hampers its use in prospective studies and real-time clinical practice [31]. A phantom study reported many (94%) handcrafted radiomic features were not reproducible and were redundant [115]. In addition, some studies concluded that the reproducibility of handcrafted radiomic features are easily affected by different acquisition and reconstruction parameters [116, 117]. Recently, the Image Biomarker Standardization Initiative (IBSI) assessed the reproducibility of handcrafted radiomic features and found a set of standardized 169 features that are deemed highly reproducible [118].

Most of the published studies were retrospective cohort studies with a limited sample size, and it can only provide low-level clinical evidence to prove the efficacy of radiomics. In order to assess the efficiency of additional prognostic and predictive benefit, radiomic features and models must be compared to typical clinical variables, so that radiomics must match precision and ease of use [119]. Finally, in actual clinical practice, patient conditions and the results of diagnostic imaging are very complex with numerous lesions and comorbidities present, and a prediction model based on a single lesion might not be able to fulfill clinical needs.

## Future direction

**Fig. 3:**
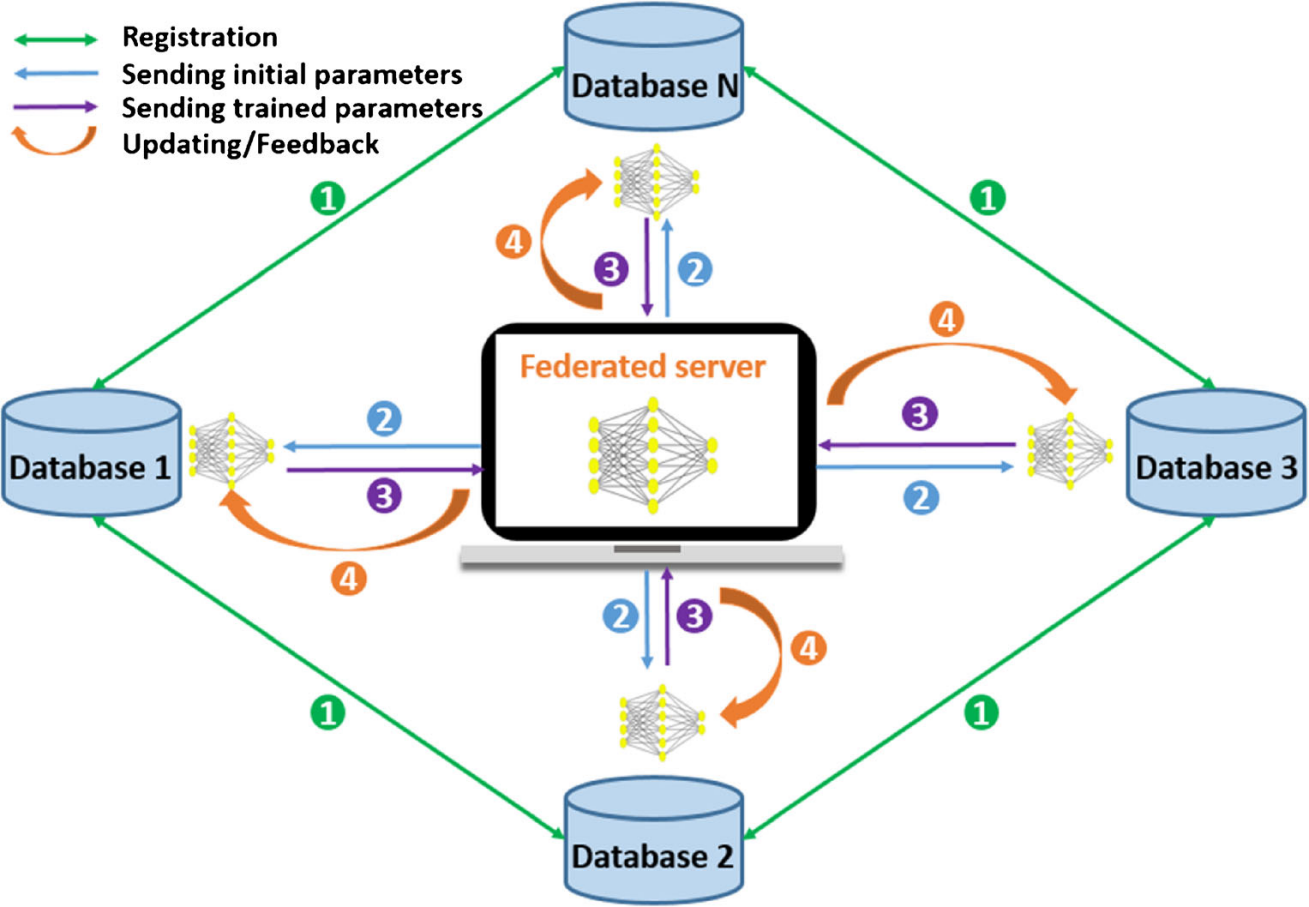
The pipeline of federated learning, which includes the main four steps: data registration among local databases, sending initial parameters to each local center from federated server, sending trained parameters to federated server from local centers, and federated server aggregates the received parameters to update local model and to give a feedback to local database

Federated learning, as a distributed machine-learning framework, can easily solve the dilemma of data silos and make it possible to integrate a model from a local database without sharing data (Fig. 3) [120, 121] . In addition, the knowledge-driven and data-driven approaches will effectively minimize reliance on big data and achieve human-machine cooperation [122].

The implementation of standardized acquisition and reconstruction protocols as well as harmonizing radiomic features will greatly improve repeatability of radiomic features and generalization of radiomic signatures. Standardization and quality evaluation of radiomics methods are crucial in the reproducibility interpretability, generalization, and long-term clinical application for any studies and trials. Elaborate prospective clinical trials with a broad sample size with high-level evidence would be required to validate the utility of radiomics. Automation, ease-of-use, and multitasking can be a one-stop solution to help physicians to accelerate clinical practice and management.

**Fig. 4:**
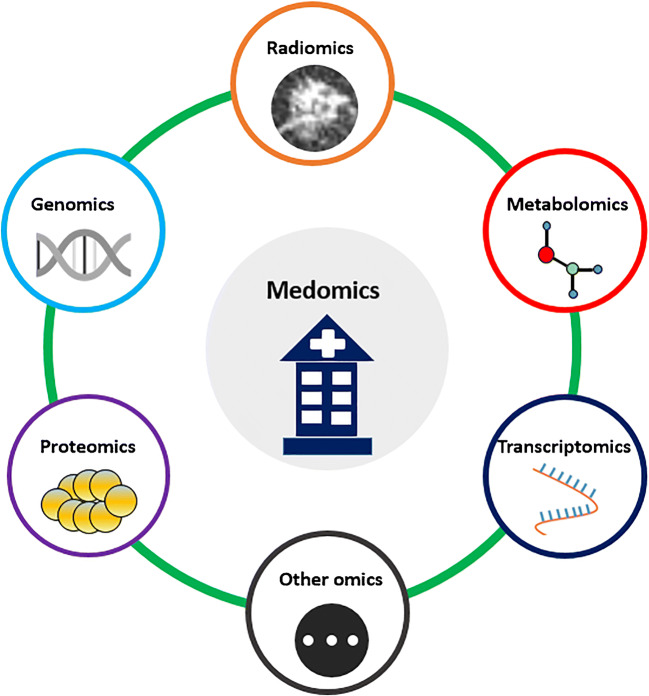
A scope of fusion of multi-discipline or multi-omics to form a “Medomics.” Other omics can be included in the Medomics, such as pathomics and lipidomics

Once existing limitations as mentioned above are overcome, investigators can use more resources to work on multi-modality fusion images and multi-discipline convergence (Fig. 4). More advanced imaging modalities and methods for lung cancer will reflect detailed structural and functional information, and will provide comprehensive and robust radiomic features and increase prospects beyond PET and CT. In addition, at a time when radiomics is taking off, AI technology is also developing rapidly in other disciplines (e.g., genomics, proteomics, and metabolomics). How to integrate radiomics with other omics to form a “Medomics” will be a fascinating avenue of further research and worth pursuing in the future.

## Conclusion

In this review, we discussed some of the latest and most impactful radiomics studies for lung cancer in the context of semantic to deep radiomic features, summarized the application of structural and functional radiomics studies, and suggested current limitations and future directions quantitative image analysis in lung cancer. Overall, radiomic approaches focused on both structural and functional images continue to evolve rapidly and are expected to bridge the gap between conventional and precision medicine. In addition, comparing and combining multi-modality functional imaging with structural imaging for lung cancer radiomics should be addressed in the future. While current challenges in data and methodology obstruct the immediate adoption of this approach in clinical practice, radiomics still holds the promise to overcome these hurdles and to be integrated in the “Medomics” workflows of the future.
